# Generation and validation of a highly sensitive bioluminescent HIV-1 reporter vector that simplifies measurement of virus release

**DOI:** 10.1186/s12977-020-00521-5

**Published:** 2020-05-19

**Authors:** James Kirui, Eric O. Freed

**Affiliations:** Virus-Cell Interaction Section, HIV Dynamics and Replication Program, Center for Cancer Research, National Cancer Institute, Frederick, MD 21702 USA

**Keywords:** Reporter virus, Nano-luciferase, Virus release

## Abstract

**Background:**

The continued persistence of HIV-1 as a public health concern due to the lack of a cure calls for the development of new tools for studying replication of the virus. Here, we used NanoLuc, a small and extremely bright luciferase protein, to develop an HIV-1 bioluminescent reporter virus that simplifies functional measurement of virus particle production.

**Results:**

The reporter virus encodes a Gag protein containing NanoLuc inserted between the matrix (MA) and capsid (CA) domains of Gag, thereby generating virus particles that package high levels of the NanoLuc reporter. We observe that inserting the NanoLuc protein within HIV-1 Gag has minimal impact on Gag expression and virus particle release. We show that the reporter virus recapitulates inhibition of HIV-1 particle release by Gag mutations, the restriction factor tetherin, and the small-molecule inhibitor amphotericin-B methyl ester.

**Conclusion:**

These results demonstrate that this vector will provide a simple and rapid tool for functional studies of virus particle assembly and release and high-throughput screening for cellular factors and small molecules that promote or inhibit HIV-1 particle production.

## Background

HIV-1 remains a major global public health challenge despite the advances made in our understanding of its replication and in the development of antiviral drugs. The HIV field continues to require innovative research tools and techniques to provide new insights on virus-host interactions that can guide the development of novel therapies.

Reporter viruses are an example of innovative tools that have been used to study HIV-1 replication. These viruses are engineered to carry reporter genes that enable the detection and quantification of virus infection and replication. An early HIV-1 reporter virus encoded a *firefly luciferase* gene in place of the *nef* gene [1]. Several other HIV-1 reporter viruses have been developed using this approach [2, 3]. With this strategy, the reporter virus infects target cells and expresses the reporter protein, allowing for detection and quantification of virus infection. However, because the reporter protein is not packaged into progeny virions, the reporter cannot be used to detect virus released from the cell. Another strategy that has been used to generate HIV-1 reporter virus involves inserting the reporter gene into the gene encoding the structural protein Gag, often between the matrix (MA) and capsid (CA) domains but in other regions of Gag as well [4–7]. Fluorescent reporter viruses made using the latter strategy have been used to detect viral transfer to target cells. A dual reporter virus encoding both a fluorescent protein-tagged Gag and a second fluorescent protein in place of *Nef* was used to study dynamics of HIV-1 replication in single cell infections [8].

The HIV-1 reporter viruses available today are highly useful tools for studying the early stages of the HIV-1 replication cycle. The early phase of the virus replication cycle begins with virus attachment and entry and ends with integration of the newly synthesized viral DNA into the host cell genome; the late phase begins with viral gene expression and culminates in virus release and maturation. Viruses that express a reporter gene (e.g., luciferase or GFP) in place of *nef* are suitable for studying the early steps of HIV-1 infection leading up to viral gene expression but not later steps. Conversely, viruses with the reporter protein embedded in the Gag protein can be used to study the late stages of HIV-1 replication. Gag-GFP virus vectors, for example, have been used to monitor Gag intracellular trafficking [5], and a Gag-YFP vector has been used to quantify virus release from cells [7]. Additional strategies have been devised to engineer reporter viruses that carry the reporter protein, allowing quantification of released virus. These include co-packaging a Vpr-GFP fusion protein with HIV-1 Gag during virus assembly, leading to the production of viruses containing Vpr-GFP [9]. Another is the expression of a membrane-anchored Gaussia luciferase upstream of the HIV-1 *nef* gene resulting in the labeling of the virus envelope with the reporter protein [10].

To enable simple and highly sensitive measurement of virus release from transfected cells, we generated HIV-1 reporter viruses in which Nano-luciferase (NanoLuc) was inserted between the MA and CA domains of Gag (Gag-iNanoLuc). NanoLuc is smaller (~ 19 kDa) and brighter (~ 150-fold) than firefly (~ 61 kDa) or *Renilla* (~ 36 kDa) luciferases [11]. We demonstrate that the HIV-1 Gag-iNanoLuc vector releases virus particles at similar levels to the WT HIV-1 molecular clone, enabling facile and highly sensitive detection of virus particle production from transfected cells. We further demonstrate the utility of the HIV-1 Gag-iNanoLuc vector as a functional tool to study HIV-1 release by using it to recapitulate disruption mediated by Gag mutations, a small-molecule inhibitor, and expression of the restriction factor tetherin. We show that although the infectivity of the Gag-iNanoLuc reporter virus is impaired, infectivity can be rescued by complementing with the WT HIV-1 molecular clone. These results highlight the potential of Gag-iNanoLuc as a tool for high throughput screening of host factors and compounds that specifically target the late stages of the HIV-1 replication cycle.

## Results

### Generation of bioluminescent HIV-1 reporter vectors

**Fig. 1:**
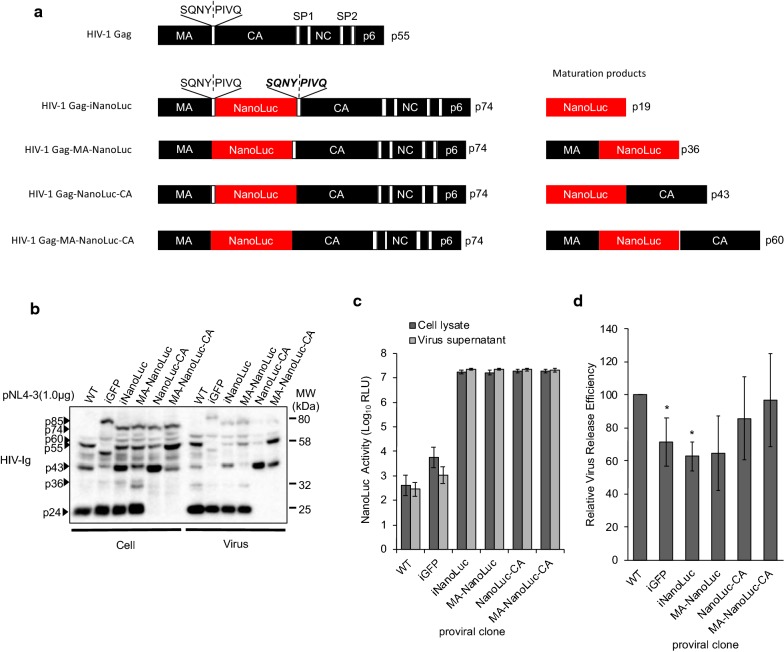
Generation of HIV-1 Gag-NanoLuc vectors. **a** A schematic representation of the HIV-1 Gag polyprotein precursor indicating different domains (black rectangles) and PR cleavage sites (white lines). The PR cleavage site between the MA and CA domains of Gag (SQNYPIVQ) is shown with the dashed line. The NanoLuc gene is represented by the red rectangle. In the HIV-1 Gag-iNanoLuc vector, the cleavage site motif is duplicated to flank the NanoLuc gene on both sides. Synonymous changes made in the duplicated coding sequence are represented in bold and italics. Maturation products of the different vectors are indicated on the right, with approximate molecular masses indicated. **b** Western blot analysis of HIV-1 Gag in lysates and supernatants from HEK293T cells transfected with the indicated HIV-1 vectors at 48 h post-transfection. Expected molecular masses of the Gag products are shown on the left; molecular mass markers are shown on the right. **c** NanoLuc activity of lysates and supernatants from HEK293T cells from (**b**) represented as Log_10_[RLUs]. **d** Relative virus release efficiency in HEK293T cells from (**b**). Error bars ± SD, n = 3 independent experiments for both **c** and **d**. *, *P *< 0.05 by one-way ANOVA compared to WT virus release efficiency

To generate an HIV-1 reporter virus that enables quantification of the reporter protein directly from virus supernatant, we employed the previously reported strategy [4, 5, 7] of introducing the reporter gene between the MA and CA domains of Gag, a location shown to tolerate genetic insertions with minimal effects on Gag protein expression and processing [4]. The NanoLuc gene was introduced between MA and CA domains of HIV-1 Gag flanked by PR cleavage sites at the N-terminus or C-terminus, or both termini of NanoLuc or not flanked by any PR cleavage site (Fig. 1a). Upon PR-mediated Gag cleavage, the resulting NanoLuc products generated from these vectors are the NanoLuc protein and MA-NanoLuc, NanoLuc-CA and MA-NanoLuc-CA fusion proteins, respectively (Fig. 1a).

To assess the effect of the NanoLuc insertion into HIV-1 Gag on Gag protein expression and PR-mediated Gag cleavage, we transfected the HIV-1 Gag-NanoLuc vectors into HEK293T cells and after 48 h, lysed the cells and pelleted virus from the supernatant. We performed western blot analysis to evaluate the expression of HIV-1 Gag-NanoLuc fusion proteins in the cells and the pelleted virus. We observed no adverse effect on Gag expression and processing and, importantly, the expected Gag PR cleavage products were generated, including some cleavage intermediates (Fig. 1b). We also measured NanoLuc activity from the cell lysate and virus supernatant and observed that all the vectors yielded robust NanoLuc activity; i.e., up to 2.0x10^7^ relative light units (RLUs) (Fig. 1c). Finally, we calculated virus release efficiency (VRE) from the same samples and found that release efficiency was modestly reduced (< twofold) compared to that of WT Gag but similar to the Gag-iGFP construct (Fig. 1d). VRE is calculated as the ratio of virion-associated p24 CA to total Gag (i.e., cellular p24 CA and Pr55Gag and viral p24 CA), normalized to WT VRE, which is set to 100. Because all four Gag-NanoLuc constructs were similar in terms of Gag expression and NanoLuc activity and displayed only modest (< twofold) differences in VRE, we chose to proceed with the pNL4-3 Gag-iNanoLuc vector for further characterization. Because pNL4-3 Gag-iNanoLuc has PR cleavage sites on both sides of the NanoLuc protein, which allows for complete processing of the Gag-iNanoLuc protein into the individual Gag domains (as is the case for WT pNL4-3), it is the most representative of the native Gag.

### HIV-1 Gag-iNanoLuc enables highly sensitive measurement of virus release

**Fig. 2:**
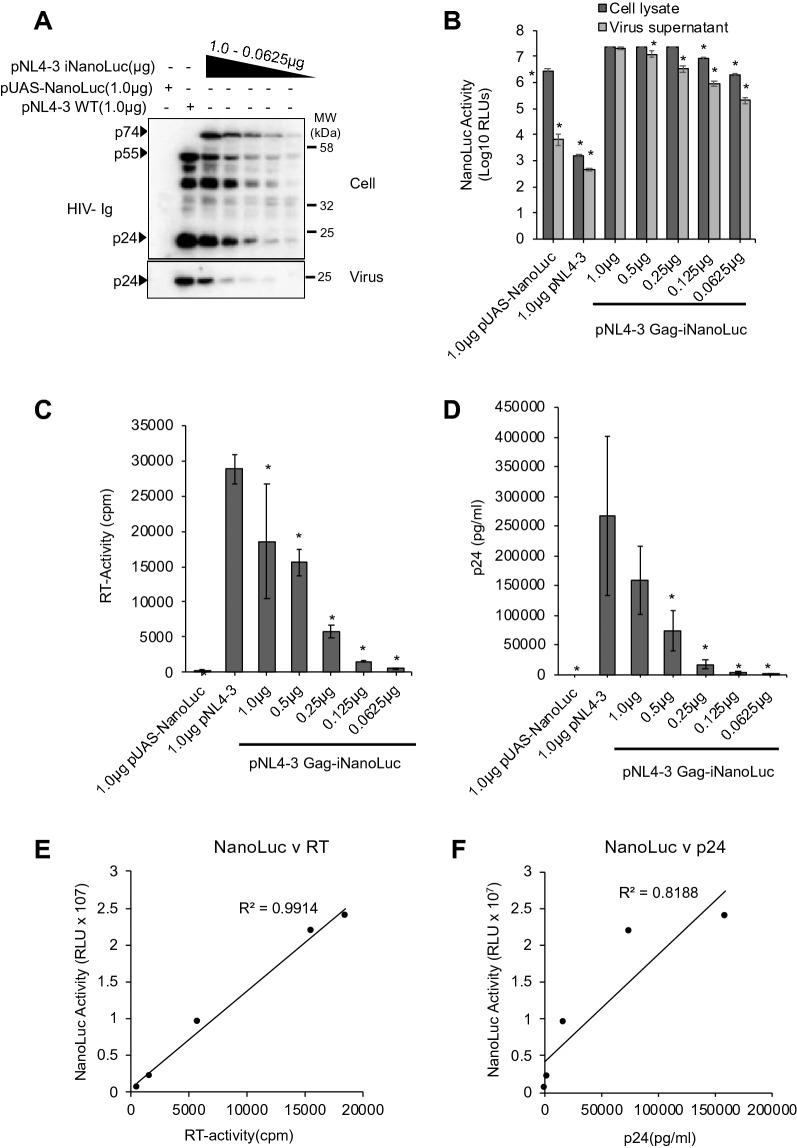
pNL4-3 Gag-iNanoLuc enables highly sensitive detection of virus release. **a** Western blot analysis of HIV-1 Gag in lysates and supernatants from HEK293T cells transfected with 1.0μg pUAS-NanoLuc, 1.0μg pNL4-3, or varying amounts of pNL4-3 Gag-iNanoLuc (1.0μg, 0.5μg, 0.25μg, 0.125μg or 0.0625μg). **b** NanoLuc activity of lysates and supernatants from HEK293T cells transfected as in **a** at 48 h post-transfection represented as Log_10_[RLUs]. **c** RT activity of supernatants from **b**. **d** Relative amounts of p24 in pg/ml in supernatants from (**b**). **e** Correlation of supernatant NanoLuc-activity and RT activity from **b**. **f** Correlation of supernatant NanoLuc activity and p24 abundance from **b**. Error bars ± SD, n = 3 independent experiments for panels **b**, **c** and **d**. *, *P *< 0.05 by one way ANOVA in panels **b**, **c** and **d** compared to 1.0μg pNL4-3 Gag-iNanoLuc in B and WT pNL4-3 RT activity and p24 in **c** and **d**, respectively

To assess the sensitivity in the detection of virus release using the HIV-1 Gag-iNanoLuc vector, we transfected HEK293T cells with either a NanoLuc expression vector, the WT HIV-1 molecular clone pNL4-3, or decreasing amounts of the HIV-1 Gag-iNanoLuc vector (i.e. 1.0, 0.5, 0.25, 0.125 and 0.0625μg). At 48 h post-transfection, we lysed the cells and purified virions from the supernatant, analyzed Gag levels by western blot (Fig. 2a), and measured NanoLuc activity from the cell lysates and supernatants (Fig. 2b). We were able to detect NanoLuc signal under all conditions tested, including at the lowest DNA input at which virion-associated Gag was undetectable by western blot. pNL4-3 Gag-iNanoLuc vector-transfected cells produced significantly higher levels of NanoLuc activity in both the cell lysate (> tenfold) and supernatant (> 1000-fold) relative to the NanoLuc expression vector control. This implies that the NanoLuc activity in the supernatant is derived from the NanoLuc protein released with the HIV-1 Gag during virus release. We also measured RT activity (Fig. 2c) and p24 protein levels (Fig. 2d) in the virus supernatant and correlated both with supernatant NanoLuc activity (Fig. 2e, f). We observed that the supernatant NanoLuc activity was positively correlated with RT activity and p24 abundance, further reinforcing the specificity of the assay.

### The defect in HIV-1 Gag-iNanoLuc particle infectivity can be rescued by co-expression with WT Gag

**Fig. 3:**
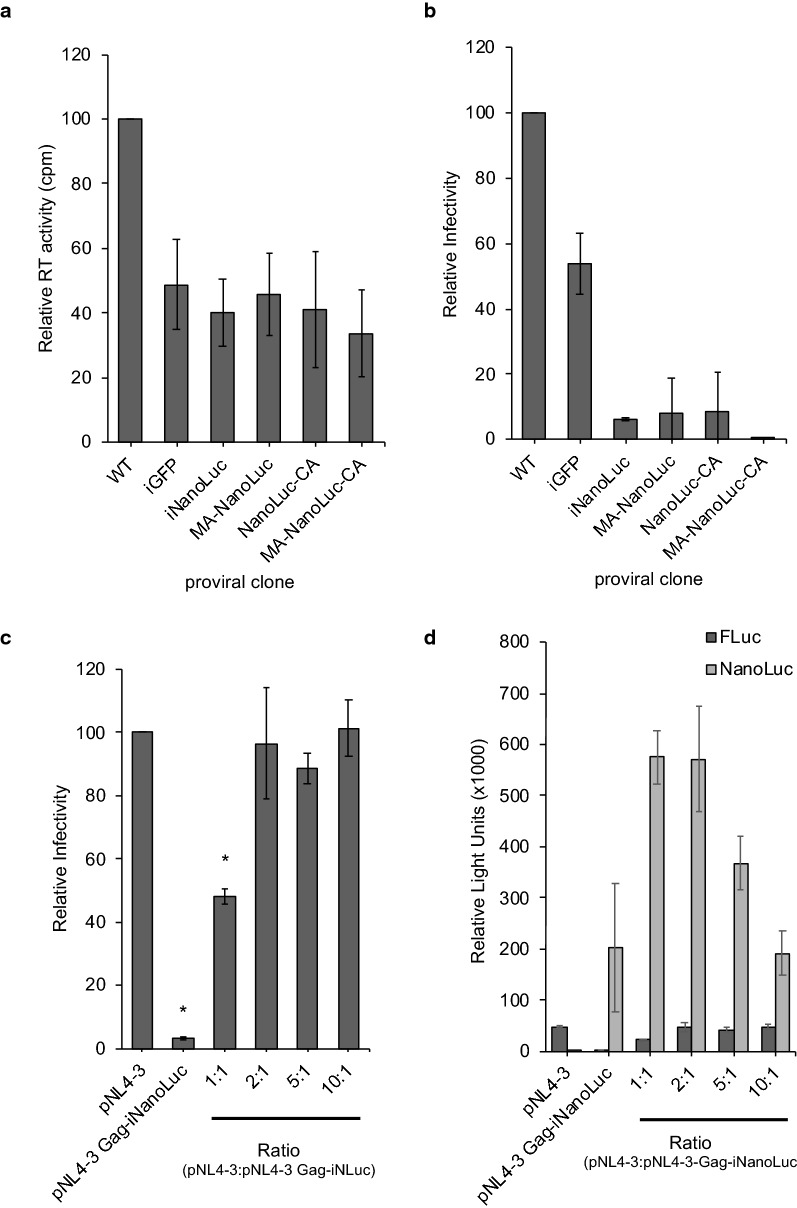
Infectivity of HIV-1 Gag-NanoLuc particles can be rescued by co-expression with WT Gag. **a** RT activity of virus particles produced in HEK293T cells transfected with either WT pNL4-3, pNL4-3 Gag-iGFP, or the indicated pNL4-3 Gag-NanoLuc vectors. RT activity is shown relative to the WT HIV-1. **b** Infectivity of virus particles from (A) in the TZM-bl indicator cells at 48 h post-infection. Viral inputs were normalized for RT activity. Infectivity is shown relative to WT HIV-1. **c** Infectivity in TZM-bl cells of virus produced in HEK293T cells transfected with either WT pNL4-3, pNL4-3 Gag-iNanoLuc, or both pNL4-3 and pNL4-3 Gag-iNanoLuc at indicated ratios of pNL4-3 to pNL4-3 Gag-iNanoLuc, with amount of total input DNA held constant. Viral inputs were normalized for RT activity. Infectivity is shown relative to WT HIV-1(pNL4-3). *, *P *< 0.05 by one-way ANOVA compared to WT pNL4-3 infectivity. **d** FLuc and NanoLuc activity from lysates of infected TZM-bl cells from **c**. Error bars ± SD, n = 3 independent experiments for all panels

We generated virus using either WT pNL4-3, the HIV-1 Gag-iGFP or the HIV-1 Gag-iNanoLuc vectors by transfecting them into HEK293T cells and collecting the supernatants containing the progeny virions at 48 h post-transfection. We quantified the relative amounts of virus in the supernatant by RT activity (Fig. 3a). We observed that RT activity in the supernatants of cells transfected with the HIV-1 Gag-iGFP and HIV-1 Gag-NanoLuc vectors was about twofold less than that of supernatants from cells transfected with WT pNL4-3. To test the infectivity of the virions produced from the HIV-1 Gag-NanoLuc vectors, we infected TZM-bl cells with the RT-normalized virus supernatants and measured the infectivity by quantifying the HIV-1 Tat-driven firefly luciferase activity (Fig. 3b). We observed that the HIV-1 Gag-NanoLuc viruses were approximately tenfold less infectious than the WT virus while the Gag-iGFP virus was only about twofold less infectious than WT virus. We also transfected the SupT1 T cell line with the HIV-1 Gag-NanoLuc vectors and monitored virus replication kinetics over several days and observed that replication was significantly impaired compared to the WT HIV-1 (data not shown). We generated viruses using the pNL4-3 Gag-iNanoLuc vector complemented with different ratios of the WT HIV-1 molecular clone pNL4-3 and tested their infectivity by measuring the HIV-1 Tat-driven firefly luciferase activity in TZM-bl cells. We observed that infectivity of the viruses generated with the pNL4-3 Gag-iNanoLuc vector was rescued when complemented with the WT pNL4-3 vector. The infectivity increased with increasing pNL4-3 to pNL4-3 Gag-iNanoLuc ratio; at ratios above 2:1 the infectivity was at WT HIV-1 levels (Fig. 3c). We also measured NanoLuc activity from the same infected TZM-bl cells and observed that the virus generated with the pNL4-3 Gag-iNanoLuc vector alone was able to express NanoLuc activity in the target cell despite being poorly infectious. The NanoLuc expression increased with increasing ratio of WT pNL4-3 to pNL4-3 Gag-iNanoLuc vector used in generating the Gag-iNanoLuc viruses. However, we observed that with higher ratios of WT pNL4-3 to pNL4-3 Gag-iNanoLuc i.e., 5:1 and 10:1, the NanoLuc expression decreased (Fig. 3d), presumably due to lower amounts of Gag-iNanoLuc genome transduced into target cells. We analyzed purified virus particles generated using the Gag-iNanoLuc proviral vectors either alone or complemented with the WT proviral clone by electron microscopy and we observed that the morphology of Gag-iNanoLuc virus particles was abnormal; specifically, the viral cores displayed a spherical shape as opposed to the canonical cone shape (data not shown). Consistent with the rescue of particle infectivity, normal, conical cores were observed when virus was generated by co-expressing the Gag-iNanoLuc vector with WT pNL4-3 (data not shown). The rescue of virion morphology by co-expressing Gag fusion proteins with WT Gag has also been observed previously [4].

### HIV-1 Gag-iNanoLuc provides a robust tool for quantifying virus release

**Fig. 4:**
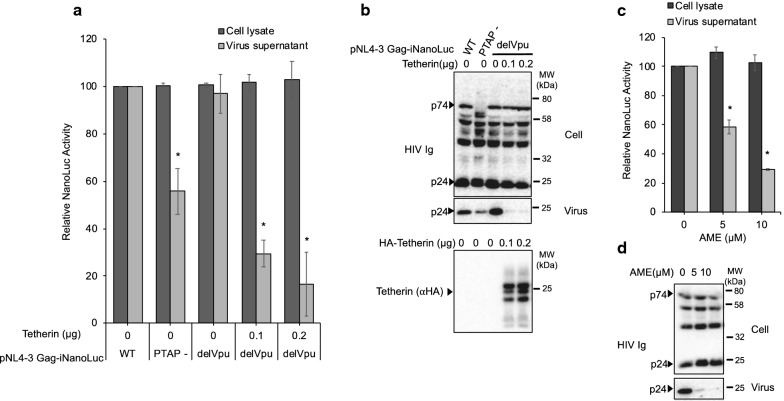
HIV-1 Gag-iNanoLuc vector recapitulates inhibition of HIV-1 particle release. **a** NanoLuc activity in lysates and supernatants from HEK293T cells transfected in 12-well plates with 1.0μg of either pNL4-3 Gag-iNanoLuc WT, pNL4-3 Gag-iNanoLuc PTAP- or pNL4-3 Gag-iNanoLuc delVpu and either 0, 0.1μg or 0.2μg HA-tetherin (BST2). NanoLuc activity in lysates and supernatants was measured at 48 h post-transfection and normalized to that of pNL4-3 Gag-iNanoLuc WT. **b** Western blot analysis of HIV-1 Gag (upper) in lysates and supernatant from **a** and HA-tetherin (lower) in cell lysates. **c** NanoLuc activity in lysates and supernatants from HEK293T cells transfected with 1.0μg of pNL4-3 Gag-iNanoLuc and treated with either 5μM or 10μM AME or vehicle at 48 h post-transfection. AME treatment was done at 24 h post-transfection following replacement of the growth media. **d** Western blot analysis of HIV-1 Gag in lysates and supernatant from **c**. Error bars ± SD, n = 3 independent experiments for **a** and **c**. *, *P *< 0.05 by one-way ANOVA in panels A and C compared to WT pNL4-3 NanoLuc activity and the 0 AME control NanoLuc activity, respectively

To examine the utility of the HIV-1 Gag-iNanoLuc vector in functional assays for virus release, we constructed versions of the vector that lacked the PTAP motif in the p6 domain of the HIV-1 Gag protein (HIV-1 Gag-iNanoLuc-PTAP-) and the *vpu* gene (HIV-1 Gag-iNanoLuc-delVpu) by cloning the iNanoLuc cassette into previously reported PTAP- and delVpu HIV-1 molecular clones [12, 13]. The p6 domain of HIV-1 Gag is required for virus release [13, 14] because of its interaction with the ESCRT machinery [15–18]. Vpu is also required for HIV-1 release in the presence of the restriction factor tetherin (also known as BST-2), which blocks release of virions by tethering them to the plasma membrane [19]. Vpu counteracts tetherin by mechanisms involving both proteasomal and lysosomal degradation and intracellular sequestration of tetherin [20, 21] We transfected HEK293T cells with the WT, PTAP- and delVpu versions of the HIV-1 Gag-iNanoLuc vector with or without varying amounts of tetherin expression vector. At 48 h post-transfection, we measured NanoLuc activity in the cell lysates and supernatants. We observed a twofold decrease in the NanoLuc activity in the supernatant of cells transfected with the PTAP- vs. the WT vector, but, as expected, no decrease in NanoLuc activity in the cell lysates. Likewise, co-transfection with a tetherin expression vector, but not an empty vector control, caused a four to tenfold decrease in NanoLuc activity in the supernatant of cells transfected with the delVpu vector. The decrease in supernatant NanoLuc activity was proportional to the amount of tetherin vector transfected (Fig. 4a, b). We performed western blot analysis of the cell lysates and the pelleted virions to analyze HIV-1 Gag expression. We observed that virus release measured by virion-associated p24 levels corresponded with the NanoLuc activity. Finally, we tested the utility of the HIV-1 Gag-iNanoLuc vector to detect impaired virus release induced by treatment of virus-producer cells with amphotericin B methyl ester (AME), a compound that inhibits HIV-1 particle production [22]. We transfected HEK293T cells with the HIV-1 Gag-iNanoLuc vector and at 24 h post-transfection treated the cells with either vehicle or increasing amounts (5μM or 10μM) of AME. At 24 h post-treatment, we collected the supernatant and measured NanoLuc activity. We observed a decrease in supernatant but not cell-associated NanoLuc activity in the presence of AME but not vehicle control. Again, the decrease in supernatant NanoLuc activity corresponded with reduced virion-associated p24 measured by western blot analysis. These results demonstrate that the Gag-iNanoLuc vector provides a highly sensitive and quantitative tool for measuring the effects of Gag mutations, host cell restriction factors, and small-molecule inhibitors on HIV-1 particle assembly and release.

## Discussion

Almost four decades since the emergence of HIV-1 and the AIDS pandemic, the virus is still a global public health concern. Numerous technical innovations have led to detailed insights into the mechanism of HIV-1 replication. However, there still remain a number of knowledge gaps, particularly regarding the late steps in the virus replication cycle, which are less amenable to high throughput analyses than the early steps. In this study, we set out to develop an HIV-1 reporter virus that would simplify and facilitate highly sensitive and quantitative measurements of the late stages of the virus replication cycle. We describe the generation of the HIV-1 Gag-iNanoLuc vector that expresses the NanoLuc reporter protein inserted into the viral Gag protein between the MA and CA domains. The Gag-NanoLuc fusion protein is expressed in the cell and released at similar levels to WT Gag, thereby enabling simple yet highly sensitive quantification of viral gene expression and virus particle production by measurement of the NanoLuc reporter protein bioluminescent activity in the cell lysates and supernatants. Importantly, we demonstrated that detection of virus release using the Gag-iNanoLuc system correlates with detection using current assays that measure supernatant RT activity or p24 abundance, and that it functionally recapitulates inhibition of virus release by Gag PTAP mutation, tetherin expression and the small molecule AME. Because of the highly sensitive nature of NanoLuc detection, we hypothesize that the Gag-iNanoLuc virus will also be useful for measuring the binding of HIV-1 particles to target cells.

The Gag-iNanoLuc vector reported here joins a number of other HIV-1 reporter vectors that are available to the field; these include Gag-fusion reporter vectors [4, 5, 7, 9], *nef*-substituted reporter vectors [1–3, 9] and virus envelope/membrane-anchored reporter vectors [10]. The Nef-substituted reporter vectors have the advantage of being replication competent, although with lesser efficiency than WT HIV-1. However, because the reporter protein is expressed only in target cells following infection and not packaged into virus particles, these vectors are suitable for detecting infection in target cells but not for measuring virus release directly from the supernatant. On the other hand, Gag-fusion reporter vectors are able to generate viruses that package the reporter protein thus allowing the quantification of virus release from cells directly from the cell culture media. Compared to reporter viruses generated by co-packaging Vpr fusion proteins with HIV-1 Gag, the Gag-iNanoLuc vector provides a higher sensitivity of detection because the NanoLuc reporter is packaged into virus particles at higher amounts than a Vpr-fused reporter, i.e., ~ 2000-3000 Gag molecules per particle vs. ~ 10-30 Vpr molecules per particle [23–25]. The high number of NanoLuc proteins that are packaged into each virus particle, coupled with the inherent brightness of the NanoLuc substrate, results in a high sensitivity of detection of virus particles in the supernatant. This allows the study of virus release dynamics at very low concentrations of the vector and thus minimizes possible cytotoxicity caused by expressing exogenous proteins and makes the Gag-iNanoLuc vector a suitable tool for functional and high-throughput assays measuring virus particle production. The high sentivity of the Gag-iNanoLuc system may lead to the detection of non-particle-associated Gag-iNanoLuc, potentially dampening the magnitude of inhibition of virus release in the presence of mutations or inhibitors relative to that calculated using less sensitive methods like p24 western blotting or RT assay (e.g., see Fig. 4). This phenomenon can be mitigated by optimizing transfection methods and the duration of transfection experiments to minimize non-specific release of non-particle-associated Gag-iNanoLuc.

Several Gag-reporter virus vectors have been reported, most of which use fluorescent reporter proteins [4, 5, 8]; these have primarily been used to study intracellular Gag trafficking dynamics and as markers of infection in target cells and not for quantification of virus particle release from producer cells. The reason for this is that fluorescent reporter proteins generally have low signal-to-background ratios due to inefficiency of the photodetector in discriminating between excitation and emission photons and also the presence of other weak fluorophores in biological samples and cell culture media [26] which renders them less suitable for quantification of released virus particles and studying virus release dynamics. Nonetheless, a Gag-reporter virus vector expressing enhanced yellow fluorescent protein (eYFP) was developed for use in quantification of virus release [7]. eYFP was found to be more suitable for this purpose than enhanced green fluorescent protein (eGFP) and enhanced cyan fluorescent protein (eCFP) as it had higher signal-to-background ratio. Because the Gag-iNanoLuc reporter vector encodes the bioluminescent NanoLuc protein, it is more suitable for quantification due to the high signal-to-background ratio of luminescence assays compared to fluorescence assays. Because luminescence relies on chemical excitation, as opposed to excitation by photons from an input light source to generate the signal i.e. emission photons, luminescence assays have very low background and hence high signal-to-background ratio. This high signal-to-background ratio results in increased sensitivity that can be as high as 1000-fold more than fluorescence assays [26]. Due to the higher signal-to-background ratio of luminescence assays, coupled with the increased brightness of the NanoLuc reporter, the Gag-iNanoLuc vector can be used at very low concentrations and exhibits a wider dynamic range. Recently, an HIV-1 NanoLuc reporter virus vector developed by inserting the NanoLuc gene upstream of the *nef* gene was reported by *Ventura* et al. [27]. This vector generates replication-competent HIV-1 NanoLuc reporter virus and was used to study HIV-1 replication dynamics in humanized mice during anti-retroviral therapy (ART). Our NanoLuc vector generates virus particles that package the NanoLuc reporter, enabling the quantification of released particles directly from the supernatant, making it a suitable tool for studying virus release.

Gag-fusion reporter vectors are generally impaired in their infectivity [4]. We found that the Gag-iNanoLuc vector was similarly poorly infectious. We also observed that the Gag-iGFP virus is more infectious than the Gag-iNanoLuc virus, despite the larger molecular mass of GFP relative to NanoLuc. We speculate that the effect of Gag insertions at the MA-CA junction on virus particle infectivity is not dependent on size alone but possibly also on how the insertion affects Gag conformation and processing. As is the case for other Gag fusion reporters [4, 7], we demonstrated that the infectivity of particles generated with the Gag-iNanoLuc vector can be rescued by complementing it with the WT HIV-1 molecular clone. We also observed that the Gag-iNanoLuc virus could express NanoLuc reporter in the target cell at 48 h following infection despite being poorly infectious. We hypothesize that the NanoLuc activity in the target cell infected with the Gag-iNanoLuc virus could be a result of packaged NanoLuc in the infecting virus particles as well as newly synthesized NanoLuc following infection.

## Conclusion

HIV-1 particle release is commonly measured by RT activity, p24 ELISA or p24 western blotting assays. These assays are relatively insensitive, laborious, expensive and time consuming, and they often involve multiple steps that can introduce errors that can compound at each step to significantly affect the final results. The HIV-1 Gag-iNanoLuc vector allows for a single-step assay to measure virus particle production and is significantly more sensitive and cheaper compared to other assays. Accordingly, this vector provides a valuable tool for studying the late stages of HIV replication, in particular in the context of high-throughput screening for late-acting cellular factors that facilitate or restrict virus release or for inhibitor screening. Previous studies have performed high-throughput screening for host factors affecting HIV-1 particle production by harvesting virus particles released from the screening/producer cells to infect target cells and measuring particle infectivity [28–30]. While this approach has generated many significant findings, a limitation is that the assay readout is the infectivity of the released particles rather than the number of virus particles per se, even though the two can often be correlated. Effects on particle assembly/release therefore cannot be distinguished from effects on infectivity. The HIV-1 Gag-iNanoLuc reporter vector simplifies virus production screening by eliminating the infection step because measurement of particle production can be performed directly from the producer-cell supernatant. This reduces the number of steps in the screening protocol and minimizes opportunities for errors and experimental variability, therefore making the HIV-1 Gag-iNanoLuc reporter vector a valuable tool for functional studies of virus release and high-throughput screening for cellular factors and small molecules that affect HIV-1 particle production.

## Methods

### Plasmids

The full-length HIV-1 molecular clone pNL4-3, a GFP-expressing HIV-1 molecular clone (pNL4-3Gag-iGFP), an HIV-1 molecular clone lacking the PTAP motif (pNL4-3PTAP-), a *vpu*-deleted HIV-1 molecular clone (pNL4-3delVpu) and an HA epitope-tagged tetherin expression plasmid (HA-Tetherin) were used in this study [5, 12, 13, 19, 31]. The *NanoLuc* gene sequence was amplified by PCR from pUAS-NLuc (Addgene plasmid no. 87696) although the origin of the sequence is from pNL1.1 (Promega, Madison, WI) [32]. The HIV-1 Gag-NanoLuc vectors were constructed by inserting the NanoLuc gene between the MA and CA domains of Gag flanked or not flanked by protease (PR) cleavage sites by overlap PCR. The PCR product generated encompassed the 5’UTR-MA-NanoLuc-CA with or without the PR cleavage sites SQNYPIVQ flanking the NanoLuc sequence. Where the SQNYPIVQ sequence was duplicated, synonymous changes were made on the DNA sequence to avoid duplicating the DNA sequence. The PCR product was then cloned in the HIV-1 molecular clone pNL4-3 via the BssHII and SpeI restriction sites.

### Cells and antibodies

HEK293T, HeLa and TZM-bl cells were cultured in Dulbecco’s modified Eagle’s medium (DMEM) with l-glutamine (Gibco), supplemented with 10% (vol/vol) fetal bovine serum (FBS), 100U/ml penicillin and 100μg/ml streptomycin at 37 °C and 5% CO_2_. TZM-bl are HeLa-derived indicator cells that express luciferase upon infection by HIV-1 [33]. SupT1 CD4^+^ T cells were cultured in Roswell Park Memorial Institute (RPMI) 1640 medium with l-glutamine (Corning, Corning, NY) supplemented with 10% (vol/vol) FBS, 100U/ml penicillin and 100μg/ml streptomycin. Pooled HIV-1 patient serum (HIV-Ig) was obtained from the NIH AIDS Reagent Program.

### Virus release assay

HEK293T cells were plated in 12-well plates and transfected with proviral plasmids using Lipofectamine 2000 (Thermo Fisher Scientific, Waltham, MA) according to the manufacturer’s protocol. At 48 h post-transfection, virus particles were purified by filtering the supernatant through a 0.45μm filter and pelleted by ultracentrifugation. Cells and virion-containing pellets were resuspended in lysis buffer [(30 mM NaCl, 50 mM Tris–HCl, pH 7.5, 0.5% Triton X-100, 10 mM Iodoacetamide, complete protease inhibitor cocktail (Roche Applied Science)]. 20ul of the cell- and virion-associated proteins were separated on 12% polyacrylamide gels by SDS-PAGE and the Gag proteins detected by western blotting using HIV-Ig antibodies. The VRE was calculated as the amount of virion-associated p24(CA) as a percentage of the total (cell- and virion-associated) p24(CA) and Pr55Gag. Where the virus release inhibitor amphotericin-B methyl ester (AME) [22] was used, HEK293T cells plated in a 12-well plate were transfected with the proviral plasmids as outlined above. The cell media were changed at 24 h post-transfection and replaced with growth media containing AME and virus particles purified at 48 h post-transfection.

### Virus infectivity assay

Viruses were generated by transfecting 2.5μg of proviral HIV-1 vectors into 0.5 × 10^6^ HEK293T cells in 6-well plates. The virus-containing supernatants were collected at 48 h post-transfection and assayed for reverse transcriptase (RT) activity as described [34]. The supernatants were used to infect 1.0 × 10^4^ TZM-bl cells per well in a 96-well plate, the volumes of the supernatants used for infection were normalized by RT activity. At 48 h post-infection, the cells were lysed in Passive Lysis Buffer (Promega, Madison, WI), and the luciferase signal was measured using Britelite Plus (PerkinElmer, Waltham, MA). Infectivity is defined as the amount of luciferase activity from the lysates of TZM-bl cells infected with RT-normalized virus supernatants [34].

### Spreading infection assay

3.0 × 10^6^ SupT1 cells were transfected with 3.0μg of HIV-1 proviral vector DNA. The cells and the DNA were mixed in 300μl of 0.8 mg/ml DEAE-dextran solution and incubated for 15 min at 37 °C. The cells were washed in 4 ml of STBS solution (25 mM Tris–HCL [pH 7.4], 0.6 mM Na_2_HPO_4_, 5mMKCL, 140mMNaCl, 0.7 mM CaCl_2_, 0.5 mM MgCl_2_), pelleted by centrifugation, resuspended in 3 ml of RPMI media (with l-glutamine and supplemented with 10% FBS, 100U/ml penicillin and 100μg/ml streptomycin) and placed in culture. The transfected cells were split every other day starting from day 3 post-transfection and aliquots of the supernatants were collected for RT activity and Nano-Luciferase activity assays.

### Nano-luciferase activity assay

To measure Nano-Luciferase activity, 15μl of cell lysates and virus supernatant (passed through a 0.45μm filter) from cells transfected with the HIV-1 Gag-iNanoLuc vectors were diluted 1:2 in cell lysis buffer. Luciferase activity was measured using the Nano-Glo assay system (Promega, Madison, WI); the Nano-Glo luciferase assay substrate was mixed with the Nano-Glo luciferase assay buffer at a 1:50 ratio and equal volume of the mix was added to the samples. The samples were incubated at room temperature for 2–3 min and luminescence was detected in a plate reader (Perkin Elmer Wallac Microbeta 1450).

### HIV-1 p24 capture immunoassay

The concentration of HIV-1 p24 in culture supernatant samples was determined by the AIDS and Cancer Virus Program using their in-house HIV-1 p24 antigen capture immunoassay.

## Data Availability

The datasets used and/or analyzed during the current study are available from the corresponding author on reasonable request.

## Abbreviations

AIDS: Acquired immunodeficiency syndrome
AME: Amphotericin-B methyl ester
ART: Antiretroviral therapy
BST2: Bone marrow stromal antigen 2
CA: Capsid
CPM: Counts per minute
DNA: Deoxyribonucleic acid
ESCRT: Endosomal sorting complex required for transport
FBS: Fetal bovine serum
GFP: Green fluorescent protein
iGFP: Internal green fluorescent protein
HA: Hemagglutinin
HEK: Human embryonic kidney
HIV-1: Human immunodeficiency virus type 1
MA: Matrix
NanoLuc: Nano-luciferase
PCR: Polymerase chain reaction
PR: Protease
RLU: Relative light unit
RT: Reverse transcriptase
SD: Standard deviation
VRE: Virus release efficiency
WT: Wildtype
